# Rare clinical case report of a contralateral effect of laser biostimulation on the bone regeneration

**DOI:** 10.1002/ccr3.5631

**Published:** 2022-03-23

**Authors:** Ghassan Habash, Soher Nagi Jayash

**Affiliations:** ^1^ Deanship of Scientific Research Al‐Quds University Al‐Quds Palestine; ^2^ Palestinian Association of Dental Implantology Al‐Quds Palestine; ^3^ School of Dentistry University of Birmingham Birmingham UK

**Keywords:** bone regeneration, contralateral effect, laser biostimulation, peri‐implantitis

## Abstract

This paper describes a unique clinical case of peri‐implantitis treated by a laser biostimulation in one side of maxilla and the effect extended to contralateral side in the maxilla. This indicates that low level laser therapy treatment may have some degree of bilateral effects within the orofacial region.

## Graphical abstract

This interesting finding calls for future studies on the bilateral effects of laser biostimulation and the clinical application of laser biostimulation with larger cohorts.

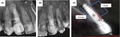

## 1 INTRODUCTION

Laser biostimulation is the application of light to increase tissue healing, reduce inflammation, and control pain. Low level laser therapy (LLLT) is used in different dental procedures, such as non‐surgical and surgical periodontal treatments, and in many clinical conditions, such as peri‐implantitis, post‐extraction and bone‐healing therapy, temporomandibular disorders, hypersensitive dentine, traumatic ulcerations, aphthous ulcers, herpes simplex virus infection of the lips, mucositis, paresthesiae, and trigeminal neuralgia.^1^, ^2^ Peri‐implantitis is an inflammatory process that affects the tissues around the implant causing bone loss. The regeneration of soft and hard tissues supporting dental implants is required to treat peri‐implantitis.^3^ The biostimulative effects of LLLT have been shown in the literature,^4^, ^5^, ^6^ and indicates LLLT can be used to enhance wound healing locally and systemically. Previously, we reported the effects of laser therapy on horizontal bone regeneration around implants.^7^, ^8^ In this case report, LLLT treatment was used to treat the bone loss around implant, surprisingly, the affected not only on the treatment side but also the non‐treatment side and indicating that there is a generalized effect within the oral region. We aimed to show a rare bilateral bone regeneration around implants treated with LLLT on one side.

## 2 CASE REPORT

### 2.1 Methods

The patient was a 20‐year‐old woman with a diagnosis of peri‐implantitis around implant placement in the upper left missing lateral incisor. The case was treated by using pontic site preservation by covering the implant with connective tissue graft and LLLT and the space restored by a minimally prepared resin‐bonded bridge. The treatment of this case was previously reported.^8^ Briefly, LLLT was applied on the surgical area using 810 nm Diode laser (QuickLase), pulsating wave mode (0.1 W) for 45 s labially and palatally to the treated implant immediately after each surgery, 7 days, 14 days 1 month, and 3 months after the surgeries. The patient was reviewed after 1 year, and cone beam computed tomography (CBCT) was taken. Written informed consent was obtained from the patient to publish this report in accordance with the journal's patient consent policy.

### 2.2 Results

**FIGURE 1:**
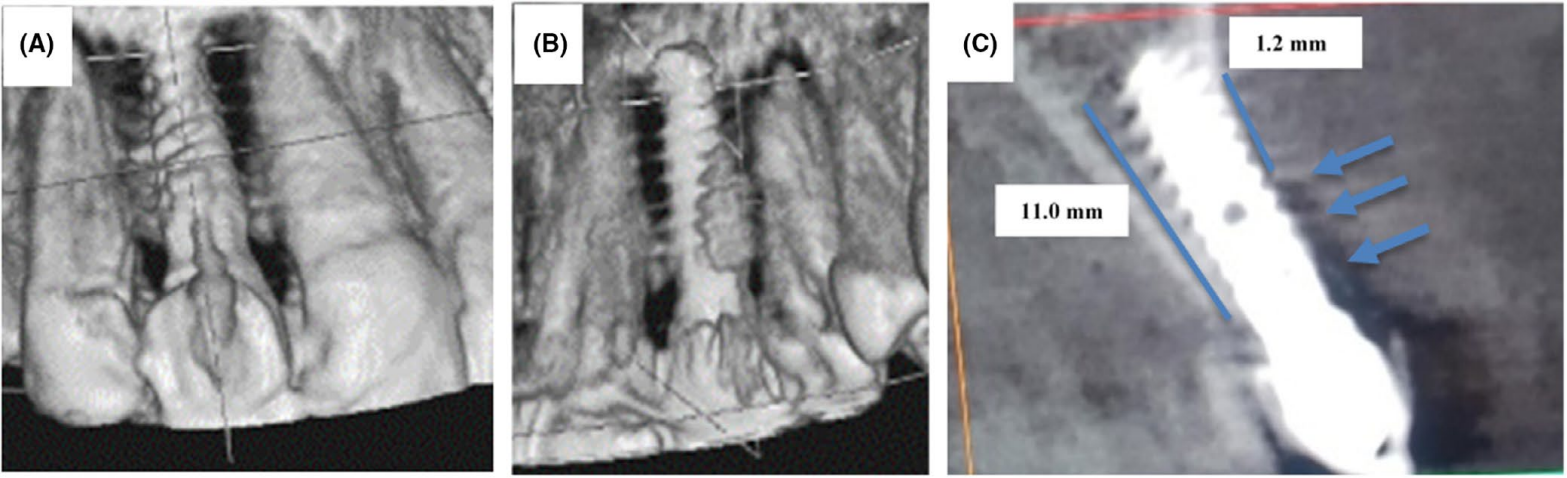
3D composition of the implant site before treatment in labial view (A) and palatal view (B). (C) The CBCT imaging of impant site showing the measurements of bone height in labioplatal view before treatment

**FIGURE 2:**
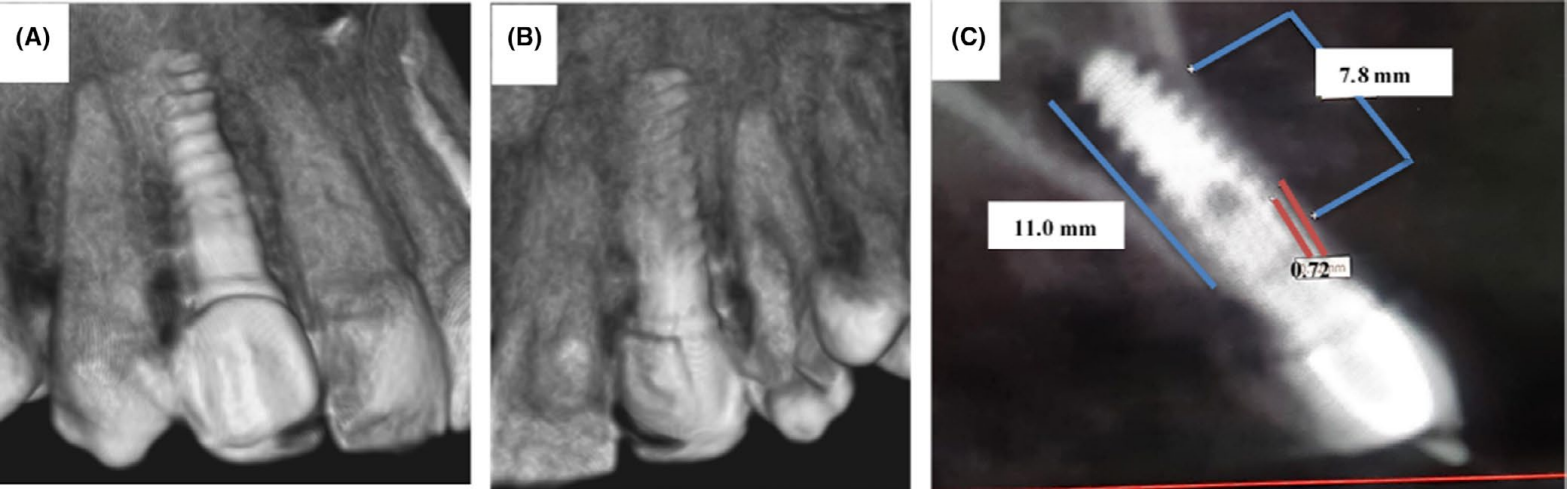
3D composition of the implant site after treatment in labial view (A) and palatal view (B). (C) The CBCT imaging of impant site showing the measurements of bone height in labioplatal view after treatment

In the first visit, clinically, the gingiva around implant was normal and there was no pain in the right lateral incisor. CBCT scan revealed bone resorption in mesial and distal surfaces and palatal surfaces around the implant (Figure 1A,B). The main complaint for the patient was in the implant in the contralateral site (left lateral incisor), so the laser treatment was not applied on the right lateral incisor. The measurement of bone before treatment was 1.2 mm in the palatal site and 11 mm in the labial site of the implant (Figure 1C). Interestingly, on 1‐year evaluation after laser treatment in contralateral site, CBCT showed bone formation around implant in contralateral site (Figure 2A,B). The alveolar bone‐height measurements from CBCT images bone were 7.76 mm in the palatal side after treatment (Figure 2C). The bone thickness adjacent to dental implant was 0.72 mm on the palatal site of the implant (Figure 2C).

## 3 DISCUSSION

The benefit of using laser around the implant over other techniques is their ability to reach hard areas around the implant to achieve better sterilization and debridement. Our previous case report describes the clinical benefits of photobiomodulation in the treatment of peri‐implantitis that reported adequate bone fill one year post‐operatively after laser therapy. Also, a previous study revealed that photobiomodulation accelerates, in vivo, the healing of the mucosa overlying alveolar sockets and can only hypothesize that also the underlying bone could reform in a shorten time.^9^

Surprisingly, the effect induced by LLLT was not restricted to the treatment side and was also observed on the contralateral side, indicating a bilateral effect in the anterior area of the maxilla. Previously, clinical study reported the application of LLLT appears to reduce the pain and sensitivity of the tooth and gingiva associated with orthodontic treatment and the analgesic effect induced by LLLT was not restricted to the treatment side and was also observed on the contralateral side, indicating an extended effect in the trigeminal area.^10^ This indicates that LLLT treatment may have some degree of bilateral effects within the orofacial region. This interesting finding will open the door for additional application of LLLT in bone healing that can be used clinically. However, this is the first clinical study reported the bilateral effect of laser in bone regeneration so this finding calls for future studies on the bilateral effects of LLLT.

## 4 CONCLUSION

The application of LLLT appears to induce bone regeneration and may have contralateral effects. Thus, the present study indicates an interesting effect of LLLT on bone formation. Further clinical applications are suggested.

## CONFLICT OF INTEREST

The authors declare there are no competing interests.

## ETHICAL APPROVAL AND CONSENT TO PARTICIPATE

Patient consent and ethics approval are not applicable.

## CONSENT

Consent for publication is signed.

## Data Availability

All supporting data are available.
